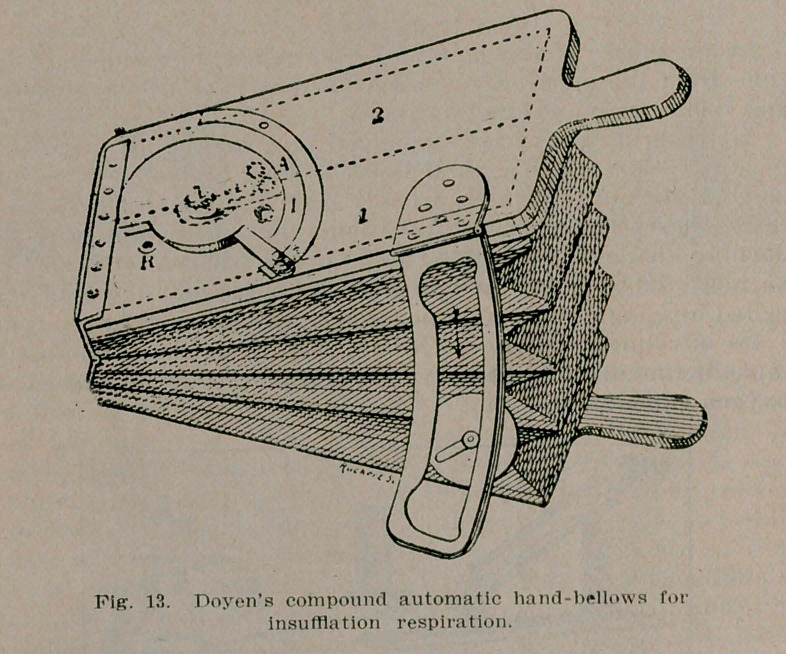# Intratracheal Insufflation

**Published:** 1916-01

**Authors:** Benjamin Merrill Ricketts

**Affiliations:** Cincinnati


### Intratracheal Insufflation.

By BENJAMIN MERRILL RICKETTS, Cincinnati.

From American Journal of Surgery, Oct., 1915. Abstract and remarks by Dr. George Edward Fell, Buffalo, N. Y.

As early as the middle of the sixteenth century Vesalius, 1514-1564, recognized the possibility of aerating the blood, after the chest had been opened, by passing a continuous current of air through the lungs.

He was requested to open the thorax of a man supposed to be dead. The heart began to beat, but he soon died. Vesalius was prosecuted for murder, but the King of Spain saved him.

Fig. 1. 1514—Vesalius—1564.

In 1667 Hook read before the Royal Society a paper entitled "An account of an Experiment Made by Mr. Hook by Preserving Animals Alive by Blowing Through Their Lungs with Bellows."

In his experiment, Hook, having laid open the entire thorax of a dog and removed the peri-cardium, sustained the animal's life, first by reciprocal inflation and deflation of the lungs in imitation of normal respiration, and then by means of a constant current of fresh air under such pressure that all respiratory movements of the lungs themselves were suspended. Both methods were successful in continuing animation and the pulmonary circulation.

Fig. 2. Fell-O'Dwyer Apparatus.

While mechanical respiration remained a valuable adjunct of laboratory experiment for almost 250 years, its failure as a human resuscitative measure in the practice of such surgeons as LeRoy de Ettioles of France, and John Hunter, Monroe and Dalrymple of England, forced the scientist, John Erichson, in 1845 to conclude that, "**In spite of all the improvement and**

Fig. 3. 1728—John Hunter—1793.

**modifications of the technique and the methods of inflation by bellows, mechanical respiration never again came into favor and was speedily forgotten when the postural methods came into use.**"

Marshall Hall was especially severe in his condemnation of forced mechanical respiration by means of bellows, and until 1887 the postural resuscitative methods of Hall, Sylvester, Schäfer, and Howard were extensively employed.

On July 23, 1887, Dr. George Edward Fell of Buffalo, N. Y., after all the postural methods of resuscitation had been tried and failed, by means of "forced respiration" saved the life of a patient who had taken twenty grains of morphine and some chloral hydrate, even after the pupils had dilated in the last stage of asphyxia. Within about three months following, Prof. Dr. Bohm of the Allgemeines Krankenhaus in Vienna, saved the life of Dr. Länger by the same method.

Other successful cases by Dr. Fell of a similar character followed, and established a new epoch of artificial respiration.

In practice Dr. Fell used an especially devised double bellows, producing a constant column of air controlled by an ingenious cornet piston valve controlling absolutely the inflation and deflation of the lungs, duplicating normal breathing. His success depended upon his long experience as a physiologist and vivisectionist, and its application from an intelligent physiological standpoint. To his valve he added an oxygen and anaesthetic tube, and **first** applied the face mask in the resuscitation of serious cases in forced respiration. In two instances —those of Dr. Williams and young Mr. Archer, reciprocal respiration was continued intermittently in the former and almost constantly in the latter during four days, with recovery of the patients, and without any untoward laryngeal or systemic complications.

Dr. O'Dwyer prepared an intubation tube connected to a single bellows with an opening, the suggestion of Fell, and by which the respirations were readily controlled.

It was through the incentive given to artificial respiration by the introduction of Fell's method of "forced respiration," and the adaptation of etherization to the technic, that the surgery of the **thorax, through its utilization became a possibility.** F. W. Parham of New Orleans was the first to successfully remove a sarcomatous growth from the walls of the thorax, using "forced respiration anesthesia" by the Fell-O'Dwyer method, for purposes of the narcosis and combating pneumothorax. The operation was performed under reciprocal breathing, and the chest walls were closed with the lungs fully inflated. Complete recovery of the patient ensued.

About 1896 the French surgeons Tuffier and Hallion concluded that their experiments justified the continuous insufflation method on man. In 1902 Matas of New Orleans turned his attention to a solution of the surgical problems involved in pneumothorax.

While Kuhn of Cassal in 1895 improved his method of insufflation by intubation and demonstrated the pulmonary application of anaesthesia personally by visits to Czerny, Trendleberg, Angerer and Lotsch, the method was not favorably received in intra-thoracic surgery on the human subject.

In 1908 Robinson of Boston intubated through the mouth to the bifurcation of the trachea, sending in air under pressure through a canula, and letting the exhaust escape by way of the remaining lumen of the trachea. He did not follow this up but became interested in progressive improvements in the Brauer and Sauerbruch methods.

Mikulicz, however, must be credited with the first systematic investigation of the physiological problems besetting intra-thoracic surgery. At his suggestion in 1903 Sauerbruch began a series of experimental researches, and in 1904 completed cabinets for intrathoracic surgery, using respectively positive or negative pressure.

Fig. 4. George Edward Fell, M. D., F. R. M. S., Buffalo, N. Y.

The outstanding pioneer of intratracheal insufflation.—Benjamin Merrill Ricketts, Cincinnati, Ohio.

The first physician to use forced respiration in actual human poisoning so far as my reading goes, was Dr. George Edward Fell.—Horatio C. Wood, Berlin Int. Med. Cong., Aug. 25th, 1890.

According to Marshall Hall and the surgeons of his day, and according to every one, **until Dr. Fell proved the contrary**, it seemed that blowing air into the delicate tissues of the lungs, must necessarily result in traumatism of this organ.—Albert J. Ochsner, Chicago Med. Socy., Feb. 2, 1910.

He (Dr. Fell) started the whole thing.—Willy Meyer, New York, 1912.

I wanted to add in discussion again my little share of admiration for the excellent and important work done and the wonderful persistency and tenacity of purpose exhibited by you, one of the very first pioneers in thoracic surgery of almost thirty years ago.—Telegram, April 28, 1915.—Willy Meyer, 1915.

Dr. Fell has done work that must be classed with the greatest inventors of any time, such as Morse, Fulton and Watt.—Discussion of Dr. Fell's paper Apr. 28, 1915.* Geo. W. Crile, Cleveland, Ohio.

Your pioneer work in artificial respiration should be adequately recognized.—(Letter) Rudolph Matas, New Orleans, La.

"If he had thrice successfully demonstrated that thousands of lives may now be saved that would have been lost six months ago, he would have been famous through the world today * * * he has not so quickly reaped cosmopolitan fame, but though it comes more slowly, it will surely come, and in his glory Buffalo will proudly share."—Editorial Jas. N. Matthews, Buffalo Express, Jan. 27th, 1888. In discussion of Dr. Albert H. Briggs, Apr. 28th, 1915. N. Y. State Med. Socy.

*Not yet published by State Society.

Fig. 5. 1805—Dr. Robert Peter—1894.

While Sauerbruch soon discarded his hyperatmospheric apparatus in favor of his negative-pressure cabinet, Brauer, working independently, developed the first positive-pressure chamber to come into general use. The apparatus devised by Karewski resembled closely that of Brauer, as did also the initial cabinets of Janeway and Green. The devices of Tiegel and Brat-Schmieden were essentially of the emergency or laboratory type, although an effort was made to adapt them to the more exacting requirements of intrathoracic surgery on the human subject.

Fig. 6. A modern type of intratracheal insufflation apparatus.

What Carrel has termed the **classical type** (cabinet) of apparatus has found its apotheosis in the new intrathoracic surgical pavilion of Willy Meyer at the German Hospital, New York, in which it is possible to operate differential, positive, or negative pressure at will.

Until the present popularization of intratracheal insufflation by the Meltzer-Auer technic, whatever real progress has been made in intrathoracic surgery on the human subject must be credited to the **classical type of** apparatus.

In 1908 Meltzer saw Sauerbruch doing intrathoracic surgery, and later witnessed some of Willy Meyer's operations at the Rockefeller Institute, and seeing both pleural cavities wide open and the animals continuing to breathe, but not trusting the evidence of his own eyes, he went into his laboratory to verify the experiments. A year later, in association with Dr. Auer, he published an article on "Continuous Respiration without Respiratory Movements." After Meltzer had perfected the technic of intratracheal insufflation in his laboratory, Elsberg, with the assistance of Yankauer, developed an apparatus for its use on the human subject, and personally administered anaesthesia by this method for a successful thoractomy performed by Dr. Lilienthal at Mt. Sinai Hospital in 1910.

Fig. 7. An old type of intratracheal insufflation apparatus, (1750) purchased by Dr. Peter in Paris in 1836, and presented by his daughter to Dr. A. H. Barkley of Lexington, Ky.

Dr. Fell had prior to this arranged his apparatus virtually in order the same as the so termed Elsberg method (see Fig. 10), and demonstrated it before the Chicago Medical Society February 2nd, 1910. "He illustrated all the points of his paper. His rubber lungs, double bellows and blower run for both positive and negative pressure by electric motor were utilized," as also the use of the three necked Woolf bottle for ether anaesthetic purposes. "In surgical cases of the thorax, his apparatus under the control of the cornet piston valve, will permit the lungs to collaspe almost completely * * * or keep the lungs completely inflated to any extent, for any length of time" * * * and the intrathoracic tube can be ntilized.

But to Elsburg, and Peck (New York) and Davies (London) belong the credit of adapting intratracheal insufflation to intratracheal surgery, but of demonstrating its value as a technic of narcosis in the surgery of the head and neck.

**Physiological Considerations.**—Progress in intrathoracic surgery has depended absolutely on the mechanical control of pneumothorax during the operative procedure. This mechanical control has veried in different methods, from that of the Meltzer-Auer technic, in which the lungs for certain periods have been so distended by a constant current of air as to preclude more or less any respiratory movements, to that of Volhard-Sollman, in which the lungs have been allowed to collapse, while a current of oxygen sustained life.

Any consideration of pneumothorax involves an understanding of some elementary facts of the respiratory mechanism. Respiration is made possible in the thorax of the human subject by a partial vacuum existing in the pleural space after the contraction of the lungs during expiration. This vacuum is represented by a varying negative pressure of from 4 to 10 mm. of mercury. Consequently thoracotomy requires either positive-pressure insufflation, with or without respiratory movements, or autorespiration in the negative or differential pressure cabinet, to prevent the collapse of the lung resulting in dyspnea, displacement of the thoracic viscera, shock and death.

Fig. 8. Original model of the Matas intratracheal insufflation apparatus.

With the body of the patient within the cabinet in which the air has been rarefied to approximate the negative pressure in the pleural cavity (4 to 10 mm.) and the head of the patient is outside the chamber, permitting the respiring of normal pressure atmosphere, Sauerbruch, by varying the negative pressure as required, has been able to conduct intrathoracic operations with almost the same confidence as in abdominal surgery. In the Meyer cabinet differential pressure allows the surgeon to use positive, negative, or combinations of both pressures as needed. The great distinction between the cabinet control of the pneumothorax and intratracheal insufflation is that in the former autorespiration is depended on to conserve life, and already overtaxed and weakened nerve centers are called upon to formulate respiratory impulses, while during intratracheal insufflation these centers can be made to remain passive, thereby adding a determining factor between success and failure.

Respiratory movements are not only concerned in the aeration of the lungs, but also contribute a factor essential to the normal maintenance of the pulmonary circulation and of considerable importance to that of the systemic.

Meltzer has found it inadvisable to use a pressure which altogether suspends respiratory movements, resulting in apnea and $CO_2$ asphyxia, and suggests that, insofar as it is possible or necessary, the lungs be periodically deflated five or six times to secure not only a more satisfactory diffusion of the air and anesthetic in the smaller bronchi and alveoli, but also to eliminate $CO_2$ accumulation, and further to preserve the stimulus of respiratory movements upon the pulmonary and systemic ciculations. In cases of open pneumothorax in which the respiratory mechanism is not paralyzed, spontaneous respirations offer the required aid to continuous intratracheal insufflation.

Fig. 9. Dr. J. D. Bloom's modification of the Fell-O'Dwyer apparatus as used by Dr. Parham in the Charity Hospital of New Orleans.

However, it is always advisable to arrange for occasional interruptions of the continuous insufflation, especially in operations in which the thorax has to be laid wide open and the posterior and inferior portions of the lungs have to be dislocated, in which condition spontaneous respirations are of no avail. The occasional deflation of the lungs insures the continuance and efficiency of the pulmonary ventilation under all circumstances. It is essential, however, that this deflation be not allowed to result in a complete collapse of the lung, for reinflation under the circumstances may leave portions of the lung atelectatio.

Physiologically, the intrinsic value of intratracheal insufflation is exemplified not only in the original work of Fell, but also in the laboratory experiments of Shaklee and Githens on the treatment of strychnine poisoning, in which, although the very centers of respiration were paralyzed, intratracheal insufflation reached the climax of its usefulness as a measure of resuscitation and the conservation of life.

And yet it must be held that intratracheal insufflation has not reached that acme of resuscitative value, or any other measure so far utilized that the rythmical method of Dr. Fell in the two longest cases on record, where the centers of respiration were paralyzed for nearly four days and the lives of the patients were saved.

Fig. 10. The Fell apparatus with oxygen· and anesthetic bottle, 1891-1910.

Meltzer has also found that anesthesia by intratracheal insufflation is far superior in many respects to the usual methods of administering ether. The anesthesia is much safer, far more readily controlled; less of the anesthetic agent is used, patients go under and come out more rapidly, and an efficient method of artificial respiration is immediately at hand to take care of untoward complications.

All investigators have found it expedient to add a tank of oxygen to their armamentarium for intratracheal insufflation. Under certain circumstances a persistent cyanosis will develop, which nothing short of oxygenation will control.

**Technic of Intratracheal Insufflation Anesthesia.**—Apparatus for intratracheal insufflation anesthesia has multiplied rapidly since the popularization of the method by Elsberg. However, all apparatus is similar in certain essentials. The source of air current may be provided by foot bellows, hand-driven or electrically-driven pumps, and tanks of compressed air. The air-current may pass directly into the ether container, or as is more advisable, is stored in a low-pressure tank or gasometer from which it passes into a Woolf bottle, to be heated and moistened, and thence by regulating valve, either directly into, or only partially through, the ether container, thereby providing for aeration pure and simple or insufflation with varying percentages of ether.

Fig. 11. The original Fell apparatus for forced respiration with oxygen tube, 1887-1891.

Also a source of oxygenation is an expedient adjunct. The tube from the apparatus connects with a mercury manometer and thence to the intubation tube.

All experimenters have concluded that for the Meltzer-Auer technic a silk-woven catheter 30 cm. long and of a diameter one-half that of the glottis, usually from 22 to 26 of the French scale, serves as the best intubation tube. It should have an opening similar to the rectal tube at the tracheal end, should be absolutely smooth and semi-rigid to prevent it being expelled by coughing or from being compressed while in position.

Its introduction is best accomplished after the preliminary introduction of narcosis by ethyl chlorid-ether or nitrous oxid-oxygen-ether anesthesia.

Fig. 12. Later working model of the Matas apparatus.

Intubation is greatly facilitated by means of either the Jackson direct laryngoscope, Fischer's modification of Hayes's instrument, or the introducer devised by Cotton. After the patient has been deeply narcotized the mouth is opened wide and so held by a gag. The head is well brought forward and the tongue pulled forward by an assistant until the opening of the larynx is brought into view. The metal guide is introduced into the opening and the intubation tube is gently pushed onward until it is seen to pass over the epiglottis into the larynx, after which the metal guide is withdrawn and the tube is pushed further into the trachea until it meets an obstruction, which is either the wall of the right bronchus or the bifurcation of the trachea. It is then withdrawn an inch and is anchored in position to special mouth gags provided for the purpose.

The distance from the incisors to the bifurcation of the trachea is from 9 to 10 inches in the infant, 12 in a child, and about 17 inches in the adult. The glottis in the adult is one-half the distance between the incisors and the bifurcation of the trachea, and Elsberg suggests making the intubation catheter accordingly to insure greater accuracy in adjusting its location.

Fig. 13. Doyen's compound automatic hand-bellows for insufflation respiration.

One-eighth to 1/4 gr. of morphine hypodermically ten minutes before the administration of ether to reduce the irritability of the larynx; or from twenty minutes to half an hour previously when preliminary anesthesia by ether is not resorted to. The induction of narcosis by intratracheal insufflation produces spasmodic coughing while the patient remains conscious.

With the intubation tube introduced to the correct position air may be heard rushing through the catheter. Spasm of the larynx may now occur for a few moments, but is of no consequence. At this juncture the tube from the apparatus is connected to the catheter with the pressure gauge of the manometer controlling the air supply at 20 mm. and the ether percentage at 50. If the lungs are not kept properly distended by a pressure of from 10 to 20 mm. Hg, the intratracheal tube is either out of position in the right bronchus or is too small and is allowing the air to escape by way of the trachea. In the first instance the tube must be retracted and in the second either a larger tube must be introduced or else slight compression of the trachea around the tube at the jugulum must be intermittently utilized. Too large a tube causes $CO_2$ accumulation and cyanosis.

Complete muscular relaxation is usually obtained with from 50 to 75 per cent. of ether, and during the course of narcosis the breathing is quiet, respirations are reduced by one-third, the face remains pink, while the veins of the forehead become prominent; the pulse usually remains full, bounding, and regular, the pupils do not dilate, and frequently the corneal reflex is active, so much so that the condition of the patient is rather one of analgesia than anesthesia. Reaction from the anesthesia is so rapid that care must be exercised to keep up etherization throughout the entire operation. The depth of narcosis is controlled by increasing the air pressure and the percentage of ether while at the same time avoiding a condition of apnea, which supervenes at pressures of from 30 to 40 mm. Cyanosis and the accumulation of $CO_2$ during the operative procedure are controlled by periodic deflation and occasional oxygenation with air. Pure oxygen is dangerous on account of its toxicity when its tension becomes too great, and it is too freely absorbed by the circulation.

At the close of anesthesia, ether is turned off, and pure air, or a combination of air and oxygen, is insufflated under slightly increased pressure to blow out the ether from the trachea and alveoli. Patients come out from under the influence of pulmonary anesthesia by this method almost as soon as the insufflation is discontinued and the tracheal tube removed.

While etherization in association with intratracheal insufflation appears to be the safest form of anesthesia for intrathoracic operations, Boothby, after using the nitrous oxid-oxygen-ether technic, has been favorably impressed with the latter method, and Willy Meyer also suggests that the innocuousness of nitrous oxid may play an important role in conserving patients under thoracotomy the additional shock of a poisonous anesthetic agent.

Major Allie W. Williams, U. S. A. Medical Corps, closed his discussion of Dr. Ricketts paper as follows:

In head, neck and chest surgery I feel that intratracheal insufflation anesthesia fills an indispensable place in the practice of surgery in hospitals in the military service.

---

**Cure of Leprosy.** Gaceta Medica de Costa Rica, Sept. 15, 1915, Dr. Teodoro Picado calls attention to the Heiser treatment of Leprosy in the Philippine Islands, by which four cases of leprosy have been cured and remained cured for a period of two years. The two first cases were vaccinated at irregular intervals with the antileprous vaccine of Clegg. All four received hypodermic injections of the following:

| | |
|---|---|
| Chaulmoogra Oil .................. | 60.00 |
| Camphorated Oil ................... | 60.00 |
| Resorcin ........................... | 4.00 |

These ingredients are mixed and dissolved by heat over a water bath, and then filtered. The injections are given weekly in ascending doses—the initial dose being 1cc increased to the point of tolerance. There is a great difference in cases as to the quantity of the mixture supported. In some cases very small doses cause fever and cardiac disturbance. In such, it seems better to give smaller doses more frequently. Many authorities have insisted on the necessity of administering strychnine coincidently with the chaulmoogra oil. No strychnine was given in these cases, which is a fact worth noting. Saline purgatives are employed frequently and it is observed that the patients who take prolonged baths recover quicker. Cases treated by the X-rays and by the oral administration of chaulmoogra oil are apt to recur. While chaulmoogra oil is the best drug we have in the treatment of leprosy, it cannot be regarded as a true specific. The treatment is of value in all forms of leprosy—tubercular, anesthetic and mixed. Many cases are cured by it, a great amelioration of all the symptoms is noted in many others, and in nearly all the cases the progress of the lesions is halted.

---

**Poisons Used in Rubber Industry.** Bulletin No. 179 of the Bureau of Labor Statistics mentions the following: litharge or lead oxid, sublimed lead or lead oxysulphate, white lead or basic carbonate, red lead, golden sulphid of antimony, aniline oil, carbon disulphid, carbon tetrachlorid, coal-tar benzol, naphtha, gasoline, benzine. Other coal tar or petroleum products are used in certain secret processes.

## Figures and Tables

**Fig. 1. f1:**
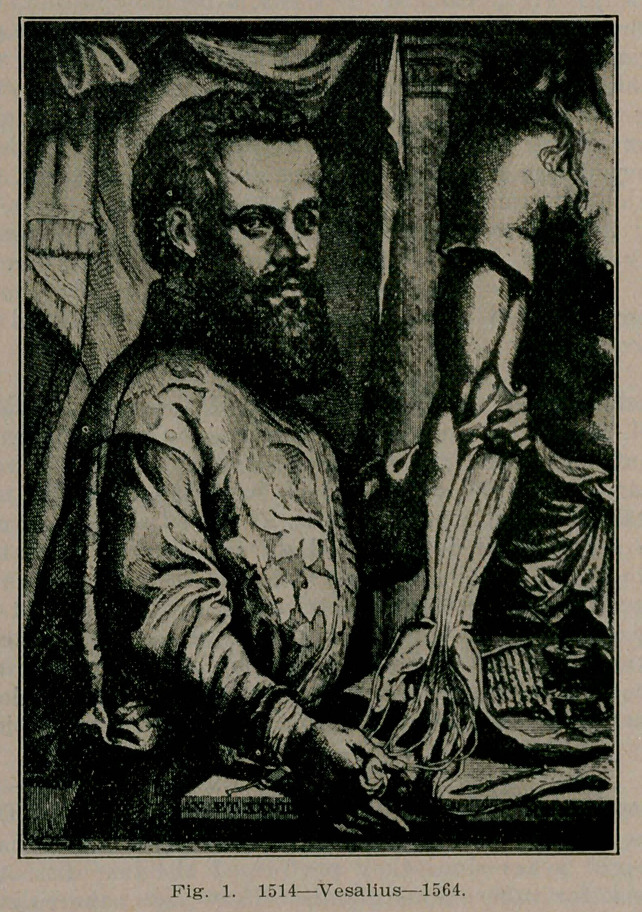


**Fig. 2. f2:**
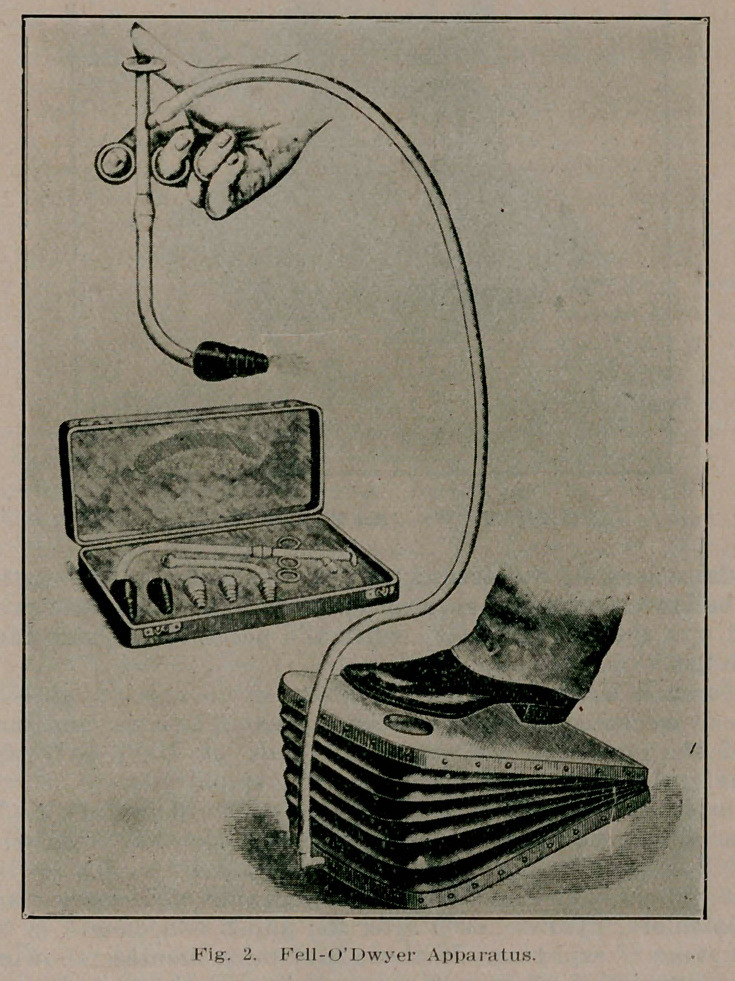


**Fig. 3. f3:**
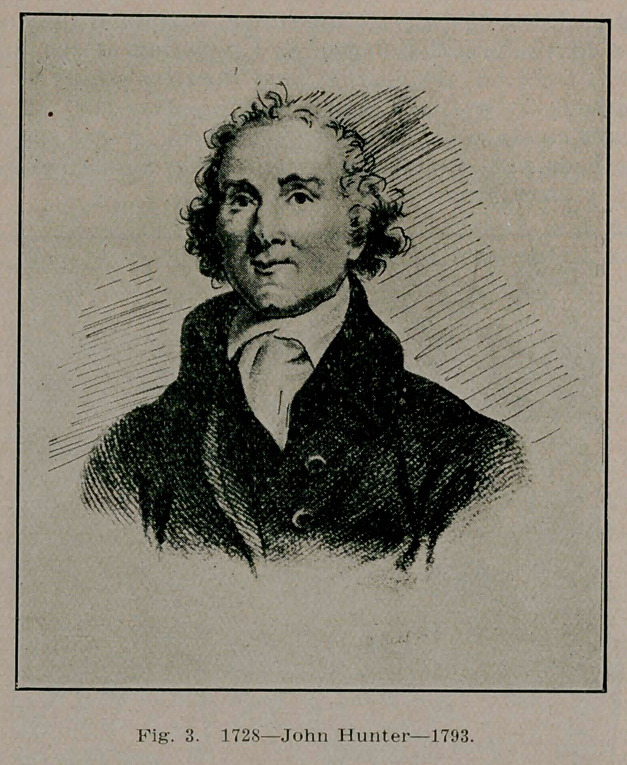


**Fig. 4. f4:**
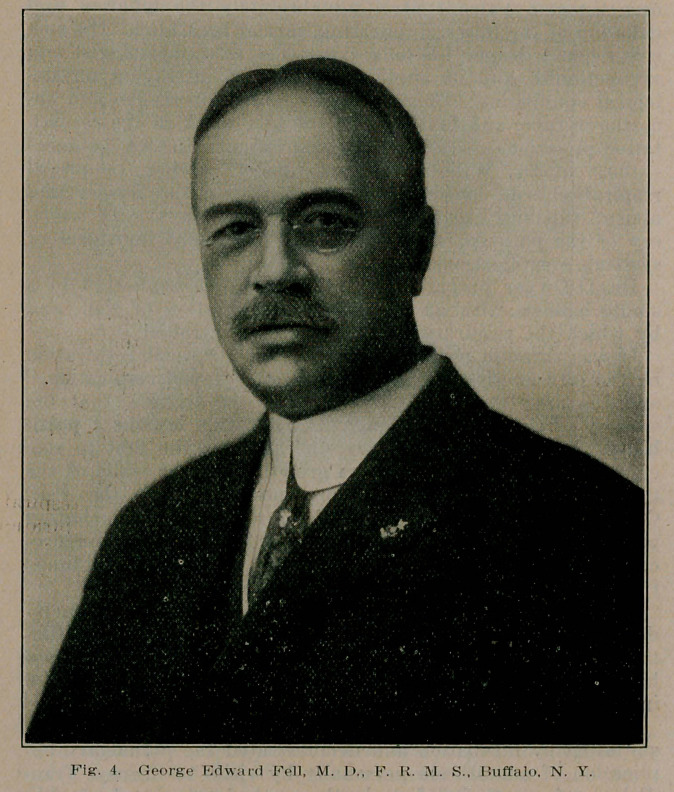


**Fig. 5. f5:**
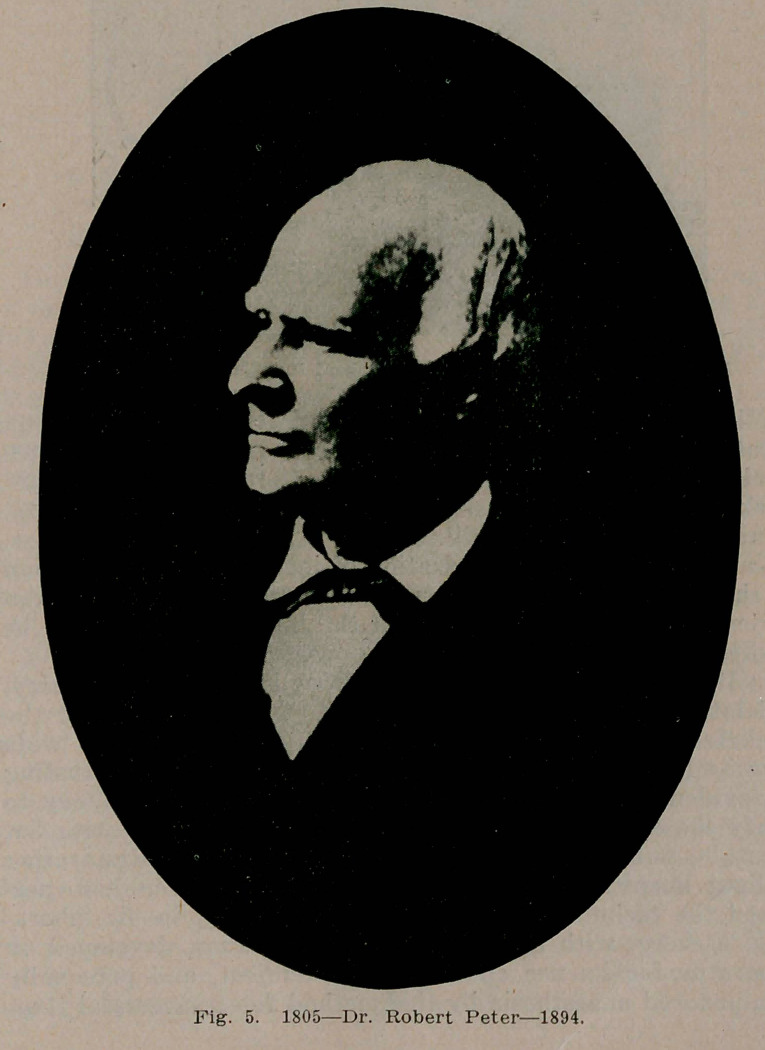


**Fig. 6. f6:**
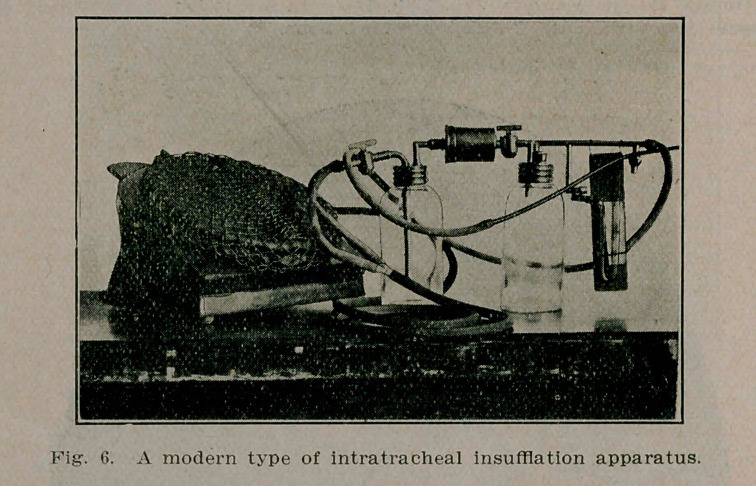


**Fig. 7. f7:**
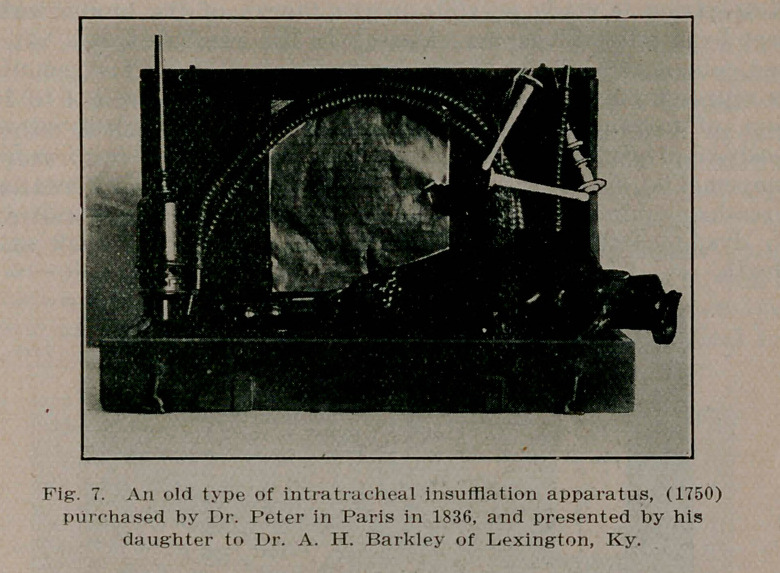


**Fig. 8. f8:**
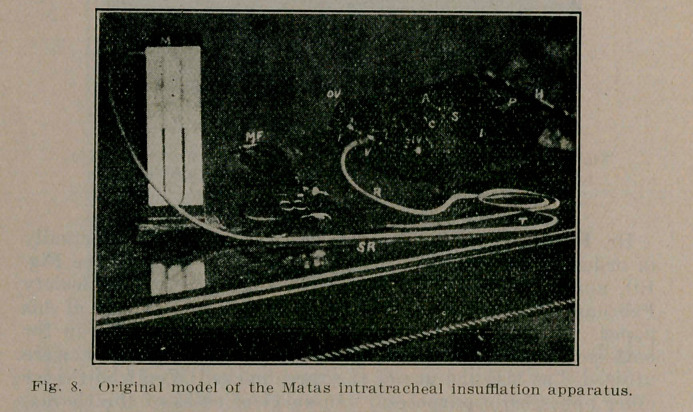


**Fig. 9. f9:**
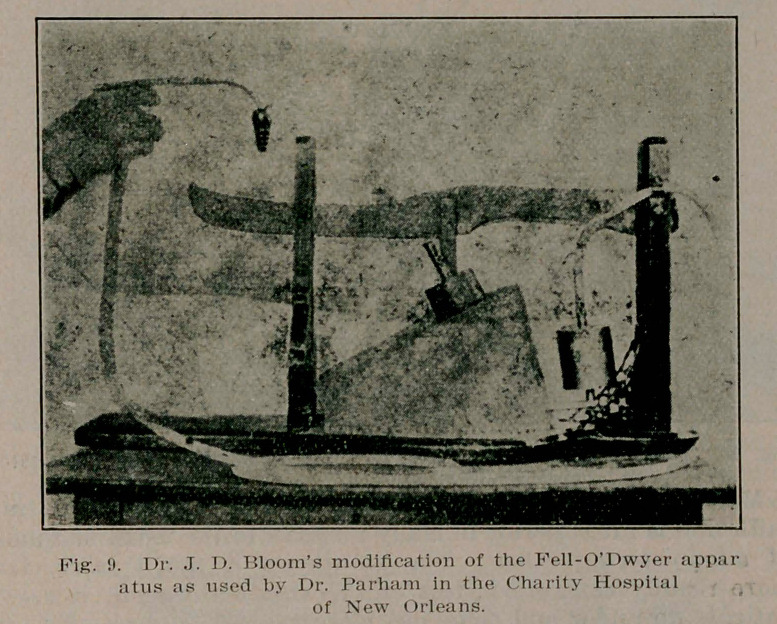


**Fig. 10. f10:**
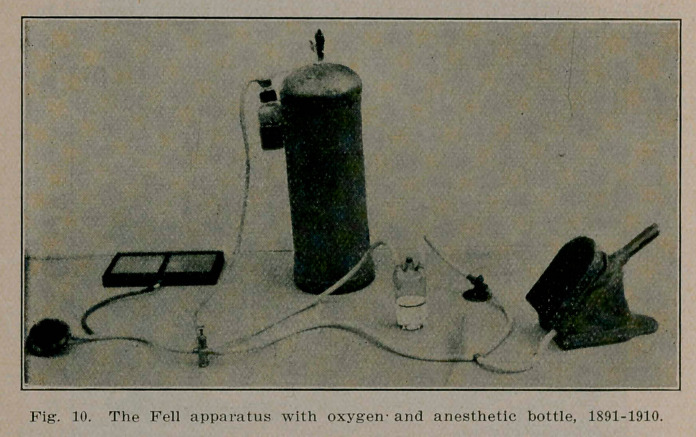


**Fig. 11. f11:**
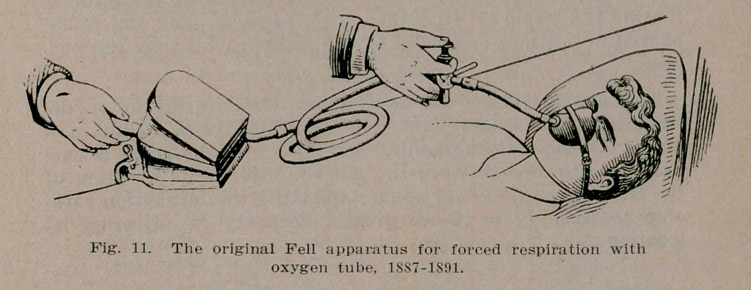


**Fig. 12. f12:**
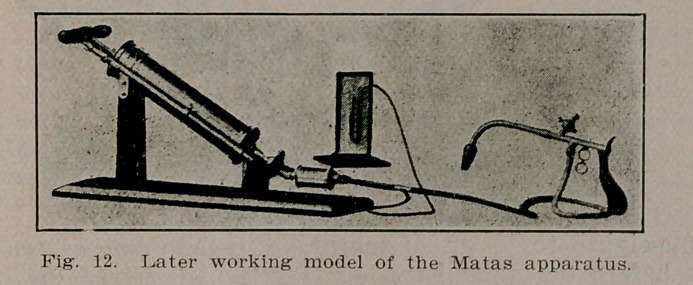


**Fig. 13. f13:**